# Serum Metabolites as Potential Biomarkers for Diagnosis of Knee Osteoarthritis

**DOI:** 10.1155/2015/684794

**Published:** 2015-03-16

**Authors:** Qingmeng Zhang, Heng Li, Zhendong Zhang, Fan Yang, Jiying Chen

**Affiliations:** Department of Orthopedics, The General Hospital of Chinese Army, Beijing 100853, China

## Abstract

Knee osteoarthritis (OA) is a highly prevalent chronic degenerative joint disease that mainly affects the elderly population. The aim of this study was to investigate serum signature metabolites as potential biomarkers for early diagnosis of knee OA. Global serum metabolic profiles of 40 patients with knee OA and 20 healthy controls (HC) were analyzed by ultra-performance liquid chromatography coupled to mass spectrometry. An OA-specific metabolic profile was established that can clearly discriminate patients with OA from HCs. Fourteen metabolites that are involved in the metabolism of amino acids, purine, energy, glycolysis, fatty acids, and lipids were significantly altered in patients with OA compared to HCs. These metabolites could be potentially used as biomarkers for the diagnosis of knee OA.

## 1. Introduction

Osteoarthritis (OA) is a highly prevalent chronic degenerative joint disease that is characterized by the progressive destruction of articular cartilage. Compared to that affecting other joints, OA of the knee is most likely to cause functional impairment, which not only contributes to reduced quality of life but also is the leading cause of joint replacement in the elderly population [1]. The reported overall incidence of knee OA is about 40% in elderly persons aged over 65 years [2]; however, this percentage is based on current diagnostic criteria that mainly rely on radiographic evaluations and clinical manifestations including pain symptoms and stiffness in the affected joints. Indeed, OA could be a silent disease for many years before the typical symptoms and radiographic changes emerge, and during this long-term subclinical stage, damage to articular cartilage may have occurred and become irreversible [3]. Thus, development of novel diagnostic markers that can reflect the destruction of articular cartilage in real time is an attractive option for early diagnosis and better treatment of OA.

Metabolomics, a recently applied approach in systems biology, focuses on the holistic investigation of endogenous metabolic responses of complex living systems and has been used in studies of both physiological and pathological conditions [4]. Disease-specific metabolic profiles have proven to be a useful marker for early diagnosis of various disorders including OA [5–8], and since metabolic perturbations occur in real time, alternations of signature metabolites could also be used in the surveillance of disease progression.

In the present study, we applied ultra-performance liquid chromatography coupled to mass spectrometry (UPLC-MS) to compare the serum metabolic profiles between patients with knee OA and healthy controls (HC). Our ultimate goal was to identify signature serum metabolites of knee OA that could be used as biomarkers for the diagnosis of OA.

## 2. Materials and Methods

The present study met the requirements of the 1975 Declaration of Helsinki and was approved by the institutional review board of the General Hospital of Chinese Army. Written informed consent was obtained from each patient included in the study.

### 2.1. Study Population

A total of 40 patients with knee OA were recruited from outpatients of our department between July 2012 and July 2013. The diagnosis of OA was based on self-reported joint pain and stiffness and radiographic evidence of structural damage (joint space narrowing with or without the presence of osteophytes) of the affected joint [9]. The exclusion criteria were the following: (1) patients with a history of other known joint disorders (e.g., trauma, autoimmunity, and bone tumors); (2) patients with a history of other known chronic disorders that could possibly influence the metabolic profiles, including metabolic syndromes, diabetes, history of corticosteroid medication, cancer, infectious diseases, and autoimmune diseases.

The Kellgren and Lawrence grade (K-L grade) of each patient was given by a radiologist and an orthopedist as the following: grade 0, no radiological changes; grade I, doubtful narrowing of joint space and possible osteophytic lipping; grade II, definite osteophytes and possible narrowing of joint space; grade III, moderate multiple osteophytes, definite narrowing of joint space, some sclerosis and possible deformity of bone contour; grade 4, large osteophytes, marked narrowing of joint space, severe sclerosis, and definite deformity of bone contour [10]. To discriminate metabolic alterations of OA at different stages, OA patients were divided into a mild group (K-L grade II or III) and a severe group (KL grade IV). The K-L grade used for analysis was the higher one in patients in which both knees were affected.

Patients with mild OA (*n* = 20) were all newly diagnosed and treatment naïve. Their serum samples were collected right after the diagnosis. Patients with severe OA (*n* = 20) were selected from those who came to our department for surgery. Their OA was diagnosed elsewhere before, but we strictly required them not to have corticosteroid or nonsteroidal anti-inflammatory drugs (NSAIDs) for at least a month before the sample collection. Twenty age- and gender-matched healthy individuals with an absence of the exclusion criteria were recruited during their routine annual medical examination in our hospital. The control individuals were given physical and radiographic exams to exclude OA after the enrollment. Serum samples were collected in all subjects before breakfast without fasting for more than 12 hours and were stored at −80°C until used.

### 2.2. Chemicals and Reagents

Methanol and acetonitrile were purchased from Merck (Darmstadt, Germany). Formic acid was obtained from Fluka (Buchs, Switzerland). Ultrapure water was prepared using a Milli-Q water purification system (Millipore Corp., Bedford, US). The remaining chemicals were of analytical grade.

### 2.3. Sample Preparation

Prior to the analysis, every 100 *μ*L of serum was mixed with 300 *μ*L of acetonitrile to precipitate the protein. The solution was centrifuged at 13,000 RPM for 15 min at 4°C, and the clear supernatant was then transferred into a vial for UPLC-MS analysis.

### 2.4. UPLC-MS Analysis

The UPLC-MS analysis used a nontargeted method as described by Qi et al. [11] on an Agilent 1290 Infinity LC system equipped with an Agilent 6520 Accurate-Mass Quadrupole Time of Flight mass spectrometer (Agilent Technologies, Palo Alto, CA, USA). The ultimate goal of a nontargeted metabolomics analysis is to detect metabolites that are significantly increased or decreased during a pathological condition. The method is not quantitative because the significantly altered metabolites can only be identified after the analysis, but quantification of a certain metabolite requires having its high-quality standard substances prepared before analysis and assayed simultaneously with the samples. Chromatographic separations were performed at 35°C on an ACQUITY UPLC HSS T3 column (2.1 × 100 mm, 1.8 *μ*m; Waters, Milford, MA, USA). The mobile phase consisted of 0.1% (volume [v]/v) formic acid (solvent A) and acetonitrile modified with 0.1% formic acid (solvent B). The optimized elution started from 2% B at 0–3 min and increased to 95% B at 3–16 min, which was maintained for 3 min, followed by an equilibrating step of 5 min. The flow rate was set to 0.35 mL/min, and the injection volume was 3 *μ*L. The autosampler was maintained at 4°C.

An electrospray ionization source was used in the positive mode. The optimized conditions were as follows: capillary voltage, 4 KV for positive mode; drying gas flow, 10 L/min; gas temperature, 350°C; nebulizer pressure, 40 psig; fragmentor voltage, 120 V; skimmer voltage, 60 V. Data were collected in the centroid mode from 100 to 1,100* m*/*z*. Potential biomarkers were further analyzed by MS/MS; the collision energy was set at 10–50 eV.

### 2.5. Data Processing and Statistical Analysis

Raw data from the UPLC-MS were converted to common data format (.mzdata) files using the Agilent Mass Hunter Qualitative software, with the threshold set to 0.1% to exclude isotope interference. The XCMS program (http://metlin.scripps.edu/download/) was used for peak extraction, alignment, and integration in a visual matrix. All detected ions in each sample were normalized to the sum of the peak area to obtain the relative intensity of metabolites. After mean-centering and Pareto scaling, the three-dimensional data matrix, including sample names, retention time, and* m*/*z* pairs, and normalized ion intensities were imported into the SIMCA-P 12.0 software package (Umetrics, Umea, Sweden) for multivariate analysis. Parameters including *R*2*X*, *R*2*Y*, *Q*2, and permutation tests were analyzed to evaluate the quality of the models. *R*2*X* and *R*2*Y* represent the fraction of the original *X* and *Y* data matrixes used to build PLS-DA model and provide an estimation of how well the model fits the *X* and *Y* data, respectively. *Q*2 represents the predictive accuracy of the model. Higher *R*2*X* and *R*2*Y* indicate that more original data are represented. Higher *Q*2 suggests better predictive ability of the model.

Quantitative clinical data were expressed as mean ± standard deviation (SD) and were compared between groups by Student's *t*-test. The relative intensity of each detectable UPLC-MS peak was normalized into a value of 0-1 and was then imported into the SPSS (version 20.0, SPSS Inc., Chicago, IL, US) for the statistical analysis. One-way analysis of variance (ANOVA) with Tukey's post hoc tests was carried out for the comparison between groups. A two-tailed *P* value of 0.05 or less was considered statistically significant. The Kyoto Encyclopedia of Genes and Genomes (KEGG) PATHWAY database (http://www.genome.jp/kegg/) was used to construct the key pathway of different metabolites.

## 3. Results

### 3.1. Clinical Parameters of Participants

Demographic and clinical characteristics of patients with OA and the controls are shown in Table 1. Serum C reaction protein (CRP) level was significantly increased in patients with OA compared to that of HCs. No differences were identified in body mass index (BMI), white blood cell (WBC) count, and creatinine between the OA and HC group.

### 3.2. Serum Metabolomics Analysis

Serum metabolic profiles were assessed in 40 patients with knee OA and 20 HCs according to our optimized UPLC-MS analysis protocol. By comparing the ion features in the assayed samples to the reference libraries, namely, the Human Metabolomics Database (HMDB) and KEGG, 106 metabolites were finally identified.

**Figure 1:**
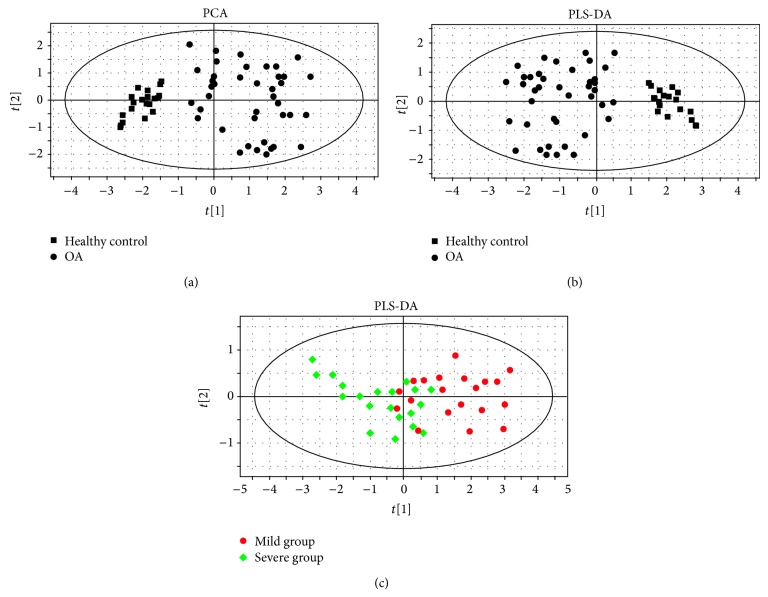
PCA (a) and PLS-DA (b) analysis based on serum UPLC-MS data showed clear tendency of separation in patients with OA and healthy controls. Further PLS-DA analysis within patients with OA showed stratification between mild and severe OA (c).

The nonsupervised multivariate analysis, principal component analysis (PCA), was performed to visualize the sample distribution and possible outliners. Score plots illustrated that patients with moderate and severe OA could be clearly separated from HCs, and no outliers were observed (Figure 1(a)). The supervised multivariate analysis, partial least squares projection to latent structures and discriminant analysis (PLS-DA), was then performed to screen the metabolites that significantly contributed to the OA-specific metabolic profile; this showed a clear tendency of separation of patients with OA from HCs (Figure 1(b), cumulative *R*2*X*, *R*2*Y*, and *Q*2 were 0.792, 0.831, and 0.663, resp.). We further demonstrated the different metabolic profiles between patients with mild and severe OA. The results showed that despite the overlap in several patients, mild and severe OA could also be stratified by our model (Figure 1(c), cumulative *R*2*X*, *R*2*Y*, and *Q*2 were 0.414, 0.469, and 0.566, resp.).

**Figure 2:**
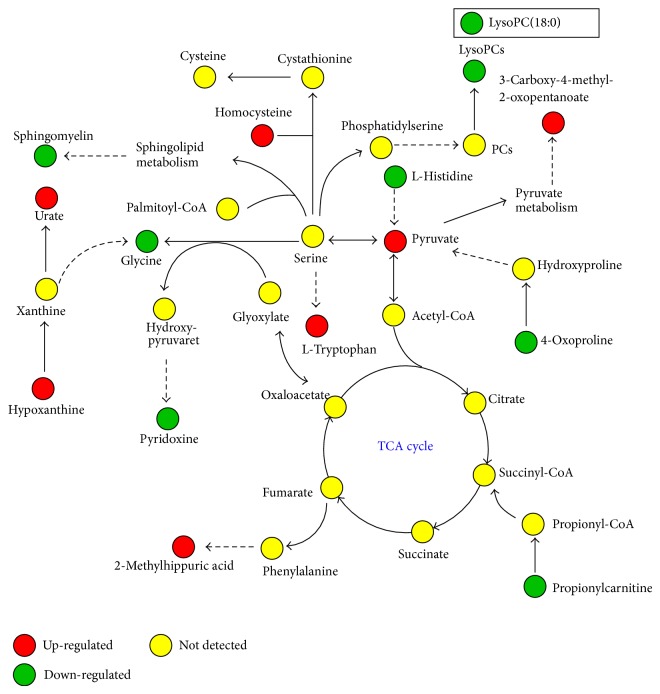
Graphic summary of the significantly altered metabolites identified and the possible metabolic pathways involved.

To identify the significantly altered metabolites that composed the signature metabolic profile of OA, ions with varying importance in the projection (>1.0) were considered as potential significantly altered metabolites and were further compared within the OA and HC groups by one-way ANOVA with Tukey's post hoc tests. Fourteen metabolites were finally identified that were significantly altered in OA and that could be used as potential biomarkers to discriminate OA patients from HCs and/or to monitor the progression of OA (Table 2).  Figure 2 illustrated a graphic summary of the significantly altered metabolites and the possible metabolic pathways involved.

## 4. Discussion

OA is considered a “wear and tear” disease that results in chronic loss of cartilage. Unlike other tissues, articular cartilage has limited metabolic activity and a weak ability to self-repair once damaged; its degeneration is generally irreversible and is accelerated by the complex local inflammatory cytokine networks [1, 12]. Thus, early interventions based on the early identification of articular cartilage destruction with methods having appropriate sensitivity and feasibility are of significant importance. Unfortunately, current laboratory exams used in OA diagnosis are either nonspecific or only effective in patients with apparent symptoms.

Metabolomics is an emerging tool based on the global study of the complement of metabolites in a biomedical sample. This feature provides the ability to map biochemical changes in the early stages of disease and hence provide potential predictive biomarkers and earlier interventions [4, 13]. In the present study, we analyzed the serum metabolomics in 40 patients with knee OA and 20 HCs to identify serum metabolic biomarkers for the early diagnosis of OA. Our UPLC-MS analysis identified an OA-specific serum metabolic profile that could clearly discriminate patients with knee OA from age- and gender-matched HCs. Moreover, the OA-specific metabolomic profile was also capable of stratifying different severities of OA, indicating its potential application for monitoring OA progression.

Our study demonstrated significantly decreased glycine and histidine levels in the serum of patients with OA compared to levels in HCs. Furthermore, serum glycine and histidine levels showed an apparent declining tendency when compared between early and late stage OA, indicating their possible role for monitoring the degree of joint damage in OA. Glycine and histidine have long been a focus in research on arthritis [14–16]. Reduced glycine and histidine in OA cartilage samples have been reported in metabolomics analyses using high-resolution nuclear magnetic resonance spectroscopy [14–16], and serum valine to histidine and leucine to histidine ratios have been proposed to be potential biomarkers for the management of OA [17]. In addition, reduced urinary glycine and histidine levels were also found to be associated with a poor response to methotrexate treatment in a collagen-induced arthritis animal model [18]. Taken together, evidence strongly suggests that glycine and histidine as well as their relevant metabolic pathways may undergo pathological alterations during the development of OA. Further explorations on the mechanisms behind these metabolic changes may provide important information on the etiology and/or treatment of OA.

Homocysteine (Hcy) is a sulfur-containing amino acid that plays pivotal roles in the biosynthesis and metabolism of multiple amino acids, nucleic acids, and phospholipids [19]. With the exception of cardiovascular diseases, homocysteine is also involved in the pathogenesis of several connective tissue disorders [17, 20–22]. Longitudinal observational studies have shown that hyperhomocysteinemia is closely associated with abnormal bone mineral density and the incidence of osteoporosis and osteoporotic fractures [22]. A higher concentration of Hcy is found in serum and synovial fluid of patients with rheumatoid arthritis (RA) [23], and Hcy level has been correlated with disease activity of patients with ankylosing spondylitis (AS) [24]. An in vitro experiment indicated that a high Hcy level is responsible for the apoptosis of osteoblastic cells via the endoplasmic reticulum stress pathway, which may support our findings that a significantly elevated serum Hcy level was observed in patients with severe OA compared to those with mild OA or HCs [25].

Serum L-tryptophan showed a significant increase in patients with OA compared to the HCs in our study. Igari and colleges reported that, unlike in RA, tryptophan metabolizes mainly through the kynurenic acid and nicotinic acid pathway in OA [26]. Kynurenic acid, as a downstream metabolite of tryptophan in OA, has been demonstrated to be altered in various human disorders and is considered to contribute to chronic inflammation. Interestingly, enhanced tryptophan metabolism was found in cultured human synovial cells stimulated by interferon-gamma, indicating its connection to the local inflammatory microenvironment in an OA affected joint [27].

Our study had several limitations. First, apart from the disease per se, metabolism can be influenced by multiple factors. In the present study, to reduce bias caused by preexisting uncontrolled conditions, we specifically excluded some common conditions from the control group that may influence the metabolic profiles. However, it has to be pointed out that this exclusion may limit the utility of our model in the clinical practice. Moreover, several of the OA signature metabolites we identified also manifested significant alternations in other connective tissue disorders, such as RA and AS [23, 24], indicating these metabolites might be more closely connected to bone destruction rather than to OA. Therefore, great challenges still exist before the eventual application of our model in the OA clinic, and further adjustments by studies with a broader disease spectrum are still needed. Second, the patient number of our study is relatively small. Further studies with larger patient numbers, ideally a prospective comparison on the longitude metabolic changes in samples of the same patients before and after the onset of OA, are necessary to build a more reliable model. Third, mild and severe OA were not clearly separated by our model, which left us a major task for further study. Besides the limited sample number, this is probably because the K-L grading system is a subjective and radiographic based method that may cause bias during the grouping of OA cases. In addition, we only studied serum samples in the present study; combined analysis of the urine and/or synovial fluid may provide superior specificity and sensitivity of our model to accomplish the goal of early diagnosis of OA.

## 5. Conclusions

By comparing serum metabolomics with UPLC-MS, we identified an OA-specific metabolic profile that could clearly discriminate patients with OA from HCs. Fourteen metabolites that involved metabolism of amino acids, purine, energy, glycolysis, fatty acids, and lipids were identified to be significantly altered in patients with OA compared to HCs. These metabolites could be potentially used as biomarkers for either the early diagnosis or progression monitoring of knee OA.

## Figures and Tables

**Table 1:** Demographic description of patients and healthy controls in the study.

	Control	OA^*^		*P* value	
		Mild	Severe	OA versus HC	m-OA versus s-OA
Number	20	20	20	NA	NA
Gender (male/female)	10/10	10/10	10/10	1.0	1.0
Age (mean ± SD)	56.3 ± 7.9	59.2 ± 8.3	57.0 ± 5.2	0.22	0.64
BMI	24.2 ± 2.3	27.5 ± 2.0	28.2 ± 3.4	0.16	0.52
CRP (mg/L)	0.7 ± 0.2	1.9 ± 0.6	2.6 ± 0.8	0.02	0.04
WBC (×10^9^/L)	4.7 ± 2.4	5.1 ± 3.1	5.4 ± 3.9	0.74	0.88
Creatinine (*μ*mol/L)	55.7 ± 21.2	58.9 ± 24.1	62.7 ± 29.6	0.84	0.79

OA, osteoarthritis; m-OA, mild osteoarthritis; s-OA, severe osteoarthritis; BMI, body mass index. CRP, C reaction protein; WBC, white blood cell.

^*^Patients with keen OA of K-L grades II and III were set as mild group and with K-L grade IV were set as severe group.

**Table 2:** Significantly altered metabolites in serum of patients with mild or severe OA compared to that of healthy controls.

	Metabolite	(*t*_*R*_/min)	(*m*/*z*)	Ion	Comparison		Related pathway
					OA versus HC	m-OA versus s-OA	
1	4-Oxoproline	0.73	130.051	[M + H]^+^	∗↓	NS	Amino acid metabolism
2	L-Glycine	0.78	120.030	[M + FA − H]^−^	∗∗↓	∗↓	Amino acid metabolism
3	L-Histidine	0.88	156.077	[M + H]^+^	∗∗↓	∗↓	Amino acid metabolism
4	Hypoxanthine	0.91	135.031	[M − H]^−^	∗∗↑	NS	Purine metabolism
5	Pyridoxine	0.96	170.082	[M + H]^+^	∗↓	NS	Energy metabolism
6	Pyruvate	1.02	133.014	[M + FA−H]^−^	∗↑	NS	Glycolysis
7	Homocysteine	1.09	136.043	[M + H]^+^	∗∗↑	NS	Energy metabolism
8	Urate	1.16	169.035	[M + H]^+^	∗∗↑	NS	Purine metabolism
9	3-Carboxy-4-methyl-2-oxopentanoate	2.85	173.045	[M − H]^−^	∗↑	NS	Amino acid metabolism
10	Methyl-hippuric acid	3.11	194.082	[M + H]^+^	∗↑	NS	Amino acid metabolism
11	L-Tryptophan	3.59	205.097	[M + H]^+^	∗∗↑	NS	Amino acid metabolism
12	Sphingomyelin (d18:1/16:0)	6.09	703.575	[M + H]^+^	∗↓	NS	Sphingolipid metabolism
13	Propionyl-L-carnitine	6.98	218.137	[M + H]^+^	∗↓	NS	Fatty acid metabolism
14	LPC (18:0)	13.11	524.372	[M + H]^+^	∗∗↓	NS	Lipid metabolism

↑: increased; ↓: decreased; ^*^ *P* < 0.05; ^**^ *P* < 0.01; NS, not significant; m-OA, mild OA; s-OA, severe OA.
